# Potential Benefits of Antioxidant Phytochemicals in Type 2 Diabetes

**DOI:** 10.3390/molecules28207209

**Published:** 2023-10-21

**Authors:** Arman Arabshomali, Shadi Bazzazzadehgan, Fakhri Mahdi, Zia Shariat-Madar

**Affiliations:** 1 Department of Pharmacy Administration, School of Pharmacy, University of Mississippi, University, MS 38677, USA; marabsho@go.olemiss.edu (A.A.); sbazzazz@go.olemiss.edu (S.B.); 2 Department of BioMolecular Sciences, Division of Pharmacology, School of Pharmacy, University of Mississippi, University, MS 38677, USA; fmahdi@olemiss.edu

**Keywords:** oxidative stress, reactive oxygen and nitrogen species, redox state, antioxidant response, diabetes, metabolic syndrome, inflammation

## Abstract

The clinical relationship between diabetes and inflammation is well established. Evidence clearly indicates that disrupting oxidant-antioxidant equilibrium and elevated lipid peroxidation could be a potential mechanism for chronic kidney disease associated with type 2 diabetes mellitus (T2DM). Under diabetic conditions, hyperglycemia, especially inflammation, and increased reactive oxygen species generation are bidirectionally associated. Inflammation, oxidative stress, and tissue damage are believed to play a role in the development of diabetes. Although the exact mechanism underlying oxidative stress and its impact on diabetes progression remains uncertain, the hyperglycemia-inflammation-oxidative stress interaction clearly plays a significant role in the onset and progression of vascular disease, kidney disease, hepatic injury, and pancreas damage and, therefore, holds promise as a therapeutic target. Evidence strongly indicates that the use of multiple antidiabetic medications fails to achieve the normal range for glycated hemoglobin targets, signifying treatment-resistant diabetes. Antioxidants with polyphenols are considered useful as adjuvant therapy for their potential anti-inflammatory effect and antioxidant activity. We aimed to analyze the current major points reported in preclinical, in vivo, and clinical studies of antioxidants in the prevention or treatment of inflammation in T2DM. Then, we will share our speculative vision for future diabetes clinical trials.

## 1. Introduction

Type 2 diabetes mellitus (T2DM) is a common hormonal disorder in the adult population, presenting in 11.3% of the US population and remaining undiagnosed in approximately 23% of adults, according to the Center for Disease Control (CDC) National Diabetes Statistics Report for 2020 cases of diabetes [1]. T2DM is caused by insulin deficiency or insulin resistance. While the incidence of T2DM in youth is increasing [2], it is rare in the pediatric population. Although most of the identified prediabetes are incidental findings without the need for intervention, some may present as clinically significant because they have a higher than normal blood sugar level or cause symptoms from medication or co-morbidities, including hypertension, hyperlipidemia, obesity, or nephropathy. 

The etiology of T2DM is diverse and appears to be due to genetic [3,4] causes and environmental factors [5]. In certain cases, however, germline, including insulin association with cancer [6,7] or somatic genetic defects [8], are associated with the development of T2DM. Risk alleles at numerous loci, such as MTNR1B, CGKAL1, and IGF2BP2, have been investigated as potentially involved in the pathogenesis, presentation of T2DM, and beta-cell function [9]. T2DM appears to be linked to dyslipidemia, atherosclerotic heart disease, and cancer [10]. Additional environmental or stochastic events in T2DM, like DNA-methylation, maternal environment [11], the impact of intrauterine [12], early infancy, and the composition and metabolic function of the gut microbiota in obese individuals [13], have also been investigated. Thus, the pathogenesis that underlies T2DM is complex and multifactorial. One of the most important components is the role of glucose autoactivation following persistently elevated circulating glucose, resulting in prooxidant production.

**Figure 1:**
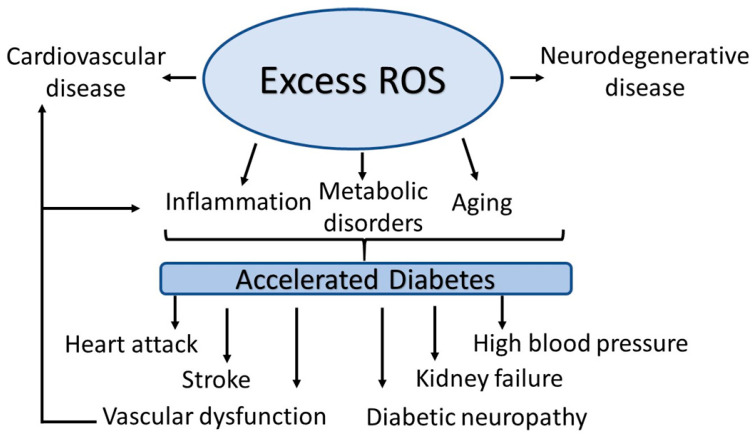
Pathological ROS in diabetes.

Oxidative stress is caused by a perturbation of prooxidants (reactive oxygen species (ROS)) and the antioxidant microecosystem that favors excess production of prooxidants relative to antioxidant defense [14]. The ROS are formed by multiple overlapping and interacting mechanisms that highlight their biological complexity and their effects on individuals’ genetic backgrounds. These enzymatic and non-enzymatic pathways include mainly oxidative phosphorylation, plasma membrane proteins such as nicotinamide adenine dinucleotide phosphate (NADPH) oxidase (NOXs), lipid metabolism within the peroxisomes, and cyclooxygenases. The abnormality of these two pathways leads to oxidative stress, which accelerates the development of diabetes complications, both microvascular and cardiovascular (Figure 1). Readers are invited to read recent reviews on this issue [15,16].

Thus, we aimed to evaluate the best evidence available on the effects of antioxidants in T2DM in order to clarify their benefits in a systemic review of the principal areas of research. PubMed, Scopus, and clinicaltrials.gov were searched using terms appropriate to each area of research. Randomized controlled trials evaluating the effect of any antioxidant on diabetes, inflammation, and antioxidant status were included. A key element of our search was to identify works that examined the impact of antioxidants, specifically in relation to oxidative stress, damage, or injury, and their capacity to scavenge ROS. Studies that reported alterations in glycemic parameters, anti-inflammatory biomarkers, oxidative stress biomarkers, and antioxidant enzyme levels were collected.

## 2. Diabetes

Diabetes is the most common chronic disease of childhood [17,18,19] and adulthood [20], and the most common type of diabetes is T2DM. While its exact cause is unknown, T2DM patients have elevated blood glucose levels, dyslipidemia, and impaired insulin receptor function [21]. There is a positive correlation between the progressive nature of T2DM and an increased risk of major cardiovascular diseases, including coronary artery disease, ischemic heart disease, stroke, and peripheral artery disease. Diabetic patients with silent cardiac left ventricular hypertrophy or renal disease are at a higher risk of developing both microvascular and macrovascular diseases [22], leading to organ and tissue damage. Diabetes-induced vascular aberration includes anatomical, structural, and functional alteration resulting in endothelial dysfunction, reduced vascular compliance, and atherosclerosis [23]. 

**Figure 2:**
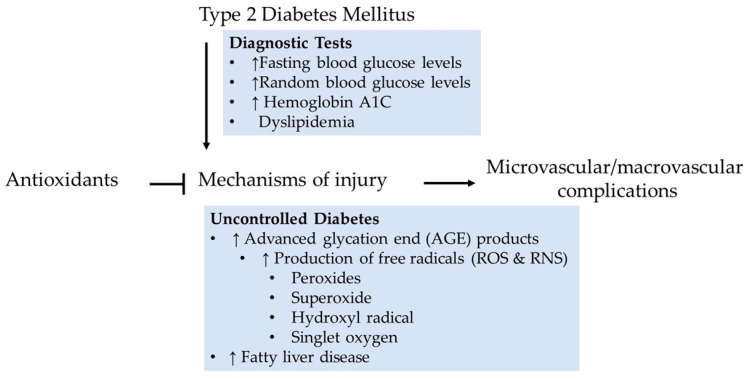
Diabetes is associated with vascular dysfunction in multiple ways. Uncontrolled free radicals are the central components of cell injury. Antioxidants may reduce or prevent ROS/RNS-induced vascular complications. ↑—indicates an increase in level.

As mentioned earlier, the cause of T2DM is unknown. But the diagnosis is made on the basis of a spectrum of clinical manifestations such as impaired regulation of hepatic glucose production, incremental decline in β-cell function, destruction of pancreatic β-cells in a small subset of type 2 diabetic patients, insulin resistance (also known as impaired insulin sensitivity), genetic mutations, dyslipidemia, or hormonal diseases. Blood glucose levels change due to diet, medications, and diabetes, a chronic heterogeneous metabolic disorder with complex pathogenesis. The above-leading clinical features are initially characterized by monitoring blood glucose to identify glucose fluctuation patterns in response to diet. A fasting plasma glucose of 126 mg/dL or more on more than one occasion is found to be associated with an increased risk of microvascular complications [24]. Elevated glucose undergoes autoactivation, which promotes the accumulation of advanced glycation end (AGE) products (Figure 2). Formed AGE products generate free radicals, including peroxides, superoxides, hydroxyl radicals, and singlet oxygen, as described in the succeeding sections. Notably, both diabetic microvascular and macrovascular complications apparently have similar etiologic characteristics. 

**Figure 3:**
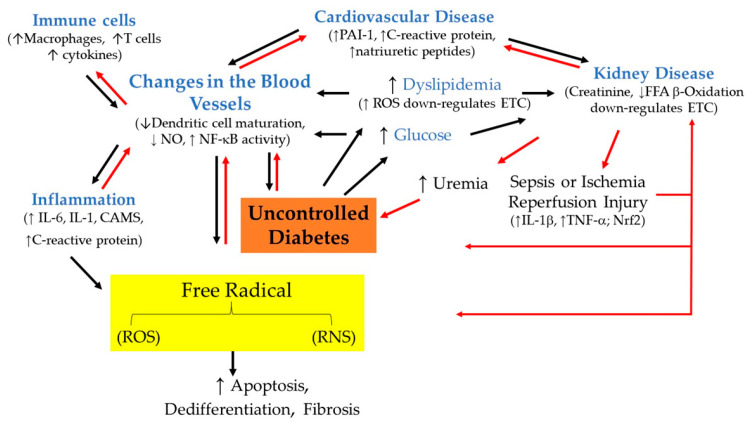
Feed-forward (black arrows) and feed-back (red arrows) events where diabetes can contribute to ROS formation. Hyperglycemia, dyslipidemia, hypertension, kidney disease, and inflammation all lead to the formation of ROS. As a consequence, elevated levels of ROS might directly contribute to fibrosis, apoptosis, and dedifferentiation, or indirectly via enhanced inflammation, reduced FFA β-oxidation, or NO bioavailability. Diabetes itself may contribute to the generation of cytokines and vascular remodeling, which can also contribute to the generation of ROS. Under normal circumstances (non-diabetic response), ROS and RNS play vital roles in numerous biological processes. ↑—indicates an increase in level; ↓—represents a decrease in level.

Insulin regulates the blood glucose levels at several key peripheral organs, highlighting a complex process. Insulin plays a major role in energy metabolism by promoting glucose uptake via glucose transporter 4 (GLUT4) translocation in skeletal muscle and adipose tissue. This increases hepatic glycogen storage and enhances lipogenesis. Evidence suggests that elevated oxidative stress blocks not only insulin secretion but also GLUT4 translocation, which leads to glucose toxicity. AGE-induced free radical production and elevated oxidative stress-induced reduction in insulin secretion and GLUT4 translocation could cause a futile cycle because it involves the continuous accumulation of glucose in the blood, which is also known as hyperglycemia (Figure 3). 

Circulating levels of free fatty acids are increased in individuals exhibiting obesity, non-alcoholic fatty liver disease, insulin resistance, or T2DM. While both growth hormone and the sympathetic nervous system promote circulating levels of free fatty acid [25], insulin reduces free fatty acid levels through suppressing lipolysis and enhancing free fatty acid clearance [26]. Chronically elevated plasma free fatty acid levels may trigger the initiation of diabetic vascular complications via increased production of oxygen-derived free radicals and the exacerbation of dyslipidemia. 

Oxidative stress plays an important role in the development of diabetes. Under physiological conditions, oxidants are neutralized by enzymatic and nonenzymatic antioxidants. Enzymatic antioxidants superoxide dismutase (SOD), catalase (CAT), and glutathione peroxidase (GPx) provide a first line of defense against free radicals to protect the integrity of biomolecules and tissues [27]. This highlights the importance of these enzymes’ expression in neutralizing excessive oxygen-derived free radicals and maintaining ROS homeostasis. While elevated levels of fatty acids cause an increase in oxidative stress through NADPH oxidative activation, the expression of the antioxidant enzymes is reduced. In addition, excessive free fatty acid liberation alters endothelium-dependent vasodilation by affecting the synthesis or degradation of nitric oxide in diabetes [28]. Thus, diabetic dyslipidemia contributes to reduced antioxidant enzyme expression and reduced nitric oxide bioavailability. 

In summary, diabetes is basically a metabolic disorder characterized by hyperglycemia, elevated free fatty acids, and insulin resistance. Each of these major hallmarks of T2DM provokes molecular mechanisms that contribute to microvascular or macrovascular dysfunction. These mechanisms include decreased bioavailability of nitric oxide, increased oxidative stress, decreased antioxidants, and activation of receptors for AGEs. The prevailing model for T2DM states that diabetes is a major risk factor for cardiovascular diseases (CVD) and is responsible for widespread morbidity and mortality. A large number of physiologic and genetic studies support this model and the central role of vascular endothelial cell metabolism [29,30,31,32], which affects the metabolism of other cell types, contributing to the pathogenesis of diabetes.

ROS are formed as natural byproducts of cellular metabolism [33] and are tightly controlled through well-coordinated cellular mechanisms. Increased ROS formation can range from low to severely high levels. At low levels, ROS act as regulators of certain signaling pathways [34] in both cell biology and physiology. At high levels, ROS cause an array of diseases, including cardiovascular, neurodegenerative, metabolic disorders, cancer, inflammation, and aging (Figure 1). ROS are involved in the degradation of pathogens and serve as intracellular signaling molecules in steady state, indicating their protective roles in infection and normal biological processes. However, their prolonged effects result in abnormal cellular physiology, which exacerbates ROS production, leading to progressive oxidative damage and the subsequent initiation of cell death. Moreover, oxidative stress-induced cell death appears to be the most proximal event of diabetes, leading to diabetic micro- and macro-angiopathic complications. Diverse components of the oxidative stress microenvironment affect cell metabolism and cell-cell homeostasis [16]. Furthermore, damage from these stressors can include mutations in mitochondrial DNA (mDNA) in patients with diabetes mellitus [35], somatic point mutations in age-related diseases [36], and immune-related somatic point mutation genes in diabetes [37]. 

The literature overflows with studies involving antioxidants. The majority of scientists in the field of experimental therapeutics for diabetes have observed that many preclinical antioxidants that are claimed to be potent and selective are often not the case due to off-target effects in general. In rare cases, the effects of antioxidants on cell function are obscure, indicating a false-positive association. In some cases, the antioxidant activity of crude extract depends on a mechanism involved in the disease process that is unelucidated. Since a close link exists among T2DM, CVD, and oxidant-induced injury, this review focuses on the recent research conducted on the effects of antioxidants on the classical biomarkers of diabetes and the components of metabolic syndrome, such as insulin resistance, atherogenic dyslipidemia, hypertension, and proinflammatory and prothrombotic states, as well as on the current biomarkers of oxidative stress, which may be useful for the treatment of diabetes.

### 2.1. Chronic Inflammation as a Cause of Diabetes 

The role of AGE/RAGE signaling in diabetes and their influence on proinflammatory signaling pathways in hyperglycemic conditions have been elaborated on in more detail elsewhere [38,39] (Figure 3). C-reactive protein (CRP) and interleukin-6 (IL-6) are significantly elevated in patients with diabetes, hypertension, and the metabolic syndrome. Those with diabetes have increased systemic levels of CRP, a protein in the blood. IL-6, a protein, is produced by numerous cells and is elevated with the severity of diabetes. Although tumor necrosis factor (TNF) alone induces the induction of CRP expression [40], the release of IL-6 in response to tissue damage is the main activator of CRP expression, which occurs in the hepatocytes. Notably, IL-1 synergistically enhances the effects of IL-6-induced CRP expression [40]. While IL-6 promotes differentiation of B-cells, it is capable of promoting cell growth in certain cells and inhibiting growth in others. This indicates that cytokines, particularly IL-6, have multiple roles to play within the body under both physiological and pathophysiological conditions (Figure 3). However, it is still unclear whether IL-6 is causing or contributing to diabetes. Both IL-6 and CRP independently predict the risk of T2DM. Interestingly, combined elevation levels of IL-6 and IL-1β in the blood are found to be the most significant predictive features for the development of T2DM [41]. Although the precise mechanism by which the proinflammatory state emerges in patients with diabetes remains to be determined, persistent hyperglycemia activates nuclear factor kappa B (NF-κB), which triggers the expression of various cytokines [42]. NF-kB not only has a prooxidant role by inducing the expression of genes such as NADPH oxidase NOX_2_ gp91phox [43], but it also has numerous anti-oxidant targets, such as manganese superoxide dismutase [44] and NF-kB-induced ferritin heavy chain upregulation via preventing iron-mediated generation of high reactive hydroxyl radicals from H_2_O_2_ [45,46]. The activation of NF-κB is mediated via ROS production [42]. Cytokines also activate NF-κB and promote the recruitment of monocytes producing M1 and M2 that cause β-cell destruction and insulin resistance [38]. The role of inflammation in diabetes pathophysiology has been reviewed in detail [47]. 

Regardless of the triggers, low-grade inflammation and vascular diseases are believed to be the principal causes of disability and premature deaths in diabetes mellitus. Dendritic cells are critical regulators of both immune and inflammatory responses. Dendritic cells sense cellular debris and modified metabolites, which are found in diabetes [48,49]. Once again, the double-edged effect of ROS raises significant concerns. Dendritic NOX2 produces low levels of ROS, which are important in promoting dendritic cell maturation and cross-presentation [50]. Damaged tissues containing dysfunctional mitochondria produce non-physiological levels of ROS. Patients with T2DM exhibit increased ROS production [31], which results in microvascular and macrovascular complications. Evidence indicates that elevated ROS inhibit cross-presentation after entering dendritic cells [51] (Figure 3). Inadequate function of dendritic cells in diabetes may feedback ROS and foster the generation of cytokines, which can contribute to the enhanced generation of ROS. Like other cells, dendritic cells are vulnerable to increased ROS levels. 

Diabetes and its clinical manifestations are characterized by chronically elevated levels of glucose and lipid, which lead to glucolipotoxic conditions. Of note, glucolipotoxicity alters mitochondrial function and, in particular, electron transport chain activity. Impaired electron transport chain activity leads to reduced and inefficient fatty acid β-oxidation [52] and promotes an increase in ROS production, triggering inflammation in highly metabolic organs including the kidneys, liver, heart, and insulin-secreting β-cells (Figure 3). The major regulators of fatty acid oxidation in these tissues with high oxidative rates are peroxisome proliferator-activated receptor a (PPARa) [53], a regulator of intra- and extracellular lipid metabolism, and AMP-activated kinase (AMPK), a sensor of cellular energy change [54]. Since both PPAR and AMPK are linked to metabolic disorders, modulation of these two molecules provides therapeutic targets for diseases such as diabetes, dyslipidemia, and inflammation. 

The current therapeutic approaches for the treatment of metabolic disease and the potential of immune-modulatory approaches have been reviewed in detail elsewhere [55]. Briefly, the oxidation of low-density lipoprotein (LDL) particles in the endothelial wall of arteries instigates monocyte infiltration [56,57]. Monocytes are then differentiated into macrophages, which accumulate oxidized lipids to form foam cells. Remarkably, oxidative stress augments intimal LDL retention and accumulation [57]. Formed foam cells stimulate macrophage proliferation and promote the recruitment of T-lymphocytes, which induce smooth muscle proliferation in the arterial walls (Figure 3). The sustained inflammatory response continues to recruit mononuclear leukocytes, both monocytes and lymphocytes. The overactive immune system and inflammatory processes [58] are driving forces in a wide variety of vascular physiological mechanisms such as endothelial activation, the loss of the endothelial monolayer due to thrombus formation and neointimal thickening [59], increased oxidative stress [57], reduced endothelial trophic effect, and disrupted blood vessel function [57,60]. It has become apparent that oxidative stress and inflammation are closely linked events, and persistent oxidative stress and chronic inflammation are now known to play predominant roles in diabetes mellitus and atherosclerosis.

### 2.2. Understanding the Relationship between Hypertension and Type 2 Diabetes Mellitus 

Statistics from the CDC and the National Health and Nutritional Examination Survey (NHANES) database show that the majority of individuals with diabetes have hypertension [61]. Dysregulation of the renin-angiotensin-aldosterone system has not only been implicated in the pathophysiology of hypertension, but it also favors T2DM [62] (Figure 3). The inhibitors of the renin-angiotensin-aldosterone system prevent diabetes in patients with hypertension. Diabetes-induced dyslipidemia and hypertension play a major role in the initiation and progression of macrovascular disease. There is a substantial overlap between diabetes and hypertension in disease mechanisms such as upregulation of the renin-angiotensin-aldosterone system and inflammation [63,64]. Should we consider diabetes and hypertension as chronic inflammatory diseases? 

The dogma suggests that inflammation is a symptom of many chronic diseases, such as diabetes, hypertension, arthritis, or Alzheimer’s disease. However, systemic reviews and meta-analyses indicate unwaveringly that chronic inflammation increases the risk of various age-related diseases, such as diabetes mellitus, hypertension, kidney disease, and rheumatoid arthritis [65,66,67,68]. 

Although it is not well clear what causes age-associated chronic inflammation, it appears that cellular senescence is a major endogenous risk factor causing chronic inflammation. Senescent cells are characterized by an arrest of cell proliferation associated with changes in cell morphology, physiology, and chromatin organization, which leads to the development of a unique phenotype known as the senescence-associated secretory phenotype [69]. An endogenous contributor to this phenotype is oxidative stress, which will be reviewed in the following section. Senescent cells are capable of secreting pro-inflammatory cytokines, chemokines, and other pro-inflammatory molecules [70]. This production leads to enhanced infiltration of immune cells and activation of other inflammatory systems, such as nitric oxide and prostaglandin. In diabetes, hyperglycemic spikes affect cytokine concentrations [71]. 

An increasing body of evidence suggests that diabetes-induced ROS production or excessive generation of ROS [72], independent of diabetes, plays a significant role in the pathogenesis of hypertension (Figure 1) [64]. Sustainedly elevated ROS levels contribute to both hypertension and diabetes. Hypertensive patients exhibit a substantial increase in plasma H_2_O_2_ levels [73]. Studies revealed the presence of decreased endogenous antioxidant enzyme activity in peripheral mononuclear cells in hypertensive patients [74] as well as in animal models of hypertension, including angiotensin II-induced hypertension [75] and nephron-vascular hypertension [76]. 

NOXs and mitochondria produce ROS under physiological and pathological conditions, as described in the following section. Angiotensin II, a vasoactive peptide, not only controls blood pressure but is also implicated in vascular ROS production. It stimulates NADH/NADPH oxidase and activates kinases, including Rho/Rho kinase [77], protein kinase C (PKC) [78], and mitogen-activated protein kinase (MAPK) [79]. Thus, ROS-induced vasoconstriction contributes to the pathogenesis of hypertension. The interplay between ROS imbalance and hypertension has been described and reviewed in detail elsewhere [80]. 

Nitric oxide (NO), a major vasodilator, is catalyzed by nitric oxide synthase. Angiotensin II and NO have opposite effects on one another [81]. The bioavailability of NO is highly dependent on its redox status [82]. The two reactive nitrogen species (RNS), namely peroxynitrite and S-nitrosoglutathione, are derived from the ^•^NO radical. Like ROS, RNS is prone to uncontrolled overproduction under stressful conditions, leading to cellular nitro-oxidative damage. The generation of ROS takes place in different compartments than that of NO generation, suggesting compartmentalization in which these molecules are tightly regulated.

In addition, other health conditions that have a key role in the development of hypertension are kidney disease, glomerulonephritis, and atherosclerosis. Remarkably, both diabetes mellitus and hypertension are responsible for chronic kidney disease [83] and adversely alter the lumen of small arteries and arterioles. Diabetic patients manifest accelerated premature vascular aging characterized by enhanced vascular smooth muscle contraction and increased vascular stiffness [84]. These structural and functional vascular changes contribute to the development of hypertension. These multiple interactive mechanisms perpetuate diabetes over time and may even cause a vicious cycle. Thus, effective management of diabetes should include a multifaceted approach to ensure adequate control of blood pressure, lipids with appropriate glycemic control [85], ROS production, RNS production, and inflammation.

## 3. The Landscape of ROS

ROS are constantly produced and inactivated by antioxidant enzymes such as catalase, superoxide dismutase, and glutathione peroxidase in biological systems. Oxidative stress has been defined as an imbalance between oxidants and antioxidants and, more recently, as a disruption of redox signaling and control [86]. It is generally accepted that oxidative stress has a fundamental role in vascular dysfunction [86]. In diabetic retinopathy, oxidative stress-induced decreased nitric oxide bioavailability contributes to endothelial cell dysfunction, basement membrane thickening, and pericyte apoptosis, resulting in altered microvasculature [87]. Lipid peroxidation is one of the major mechanisms of oxidative damage [88]. There is considerable evidence that hyperglycemia represents the main cause of complications of DM, and oxidative stress resulting from increased generation of ROS plays a crucial role in their pathogenesis [88]. The disruption of antioxidant equilibrium and elevated lipid peroxidation mechanisms for vascular damage associated with T2DM have been extensively studied. 

### 3.1. Sources of Free Radicals in the Cells 

ROS and RNS are free radicals [89] derived from both exogenous and cellular sources. The history, chemistry, and biochemistry of both ROS and RNS are reviewed in detail elsewhere [90]. ROS are reactive molecules that are derived from oxygen and are typically involved in a biological process as free radicals. These free radicals include superoxide (O_2_^•−^), hydroxyl radical (OH^•^), or non-radicals (H_2_O_2_). Like ROS, RNS are products of normal cellular metabolism. However, RNS are derived from nitrogen and can be free radicals (peroxynitrite (ONOO-) or nitric oxide (NO^•^)). While hydroxyl radical is the most reactive species that is responsible for the induction of cytotoxicity, both nitric oxide and hydrogen peroxide are less reactive. Overproduction of hydroxyl radicals and peroxynitrite can lead to impaired normal physiological redox-regulated functions and subsequent unregulated cell signaling, followed by apoptosis via lipid peroxidation, thus disrupting the integrity of the cell membrane [91] and altering lipoprotein structures.

In diabetes, although free radicals are mainly produced in mitochondria, they are also produced by other cellular organs such as the peroxisome and endoplasmic reticulum [92]. Deciphering the molecular mechanisms through which ROS directly interact with pivotal signaling molecules and the physiology of cellular ROS by mitochondria and their signaling molecules in cell proliferation and survival are of utmost importance to understand how elevated levels of ROS may result in irreparable cell damage. ROS-modulating approaches have been used to lower the excessive production of mitochondrial ROS, which is considered to be a central mechanism for the development of diabetes complications. Readers are invited to read recent reviews on this issue [15,93]. 

#### 3.1.1. Mitochondria

The biological significance of mitochondria, with special emphasis on the ROS, has already been summarized [94]. Compared to other cellular organelles, mitochondrial functioning is determined by mitochondrial DNA and the genome in the nucleus. Mitochondria have now emerged as multifaceted centers responsible for the site of energy production, the formation of ROS, and, notably, as an intracellular sink for ROS [95]. Needless to say, the mechanism by which the mitochondria perform a sink function remains unresolved. Growing evidence indicates mitochondria-related mechanisms in the pathologic process. Mitochondrial defects are also found in pathological studies of chronic diseases and major neurodegenerative diseases. The homeostasis of mitochondria is disrupted in several diseases, including atherosclerosis, renal diseases, autoimmune disorders, pancreatitis, diabetes, and chronic inflammation. In these diseases, dysfunction of mitochondrial complex I could potentially favor overproduction of ROS [96] beyond mitochondrial antioxidant capacity. The mitochondrial defects found in diabetes include fragmentation [97], fusion and fission [98], morphological changes [99], elevated mutation rates in mitochondrial DNA [100], changes in permeability of mitochondrial membranes [101], fluctuations in redox potential [102], the buildup of mutant proteins [103], and impaired oxidative phosphorylation [104]. Mitochondrial dysfunction and oxidative stress have been implicated in the pathogenesis of diabetes [97]. While autophagy is important for maintaining a balanced cell life, dysregulated or reduced autophagy [105,106,107] has been linked to diabetes-induced abnormal fuel metabolism. Based on these findings, a series of therapeutic methods targeting mitochondrial proteins and processes have been developed from bench to bedside. Mitochondria plays a pivotal role in ROS metabolism. However, mitochondrial dysfunction is not considered a hallmark of T2DM, diabetic nephropathy, or diabetic neuropathy.

#### 3.1.2. Peroxisome

Peroxisomes serve as a sensing mechanism to sense the accumulated cellular free fatty acids and activate the signaling pathway and coupling of free fatty acid degradation with that of ROS [108]. While both mitochondria and peroxisomes are involved in hydrogen peroxide formation, hydrogen peroxide is produced by leaks of electrons from donor redox centers of the mitochondrial electron transport chain [109]. Unlike mitochondria, peroxisomes transfer electrons from various metabolites to oxygen, leading to the production of H_2_O_2_ within their respiratory pathway. Since this pathway is not coupled to oxidative phosphorylation to produce ATP, its free energy is released in the form of heat. In addition, peroxisomes are an important source of O_2_^•−^, ^•^OH, and NO^•^ via various ROS-generating enzymes [110], which play a key role in signaling pathways and are responsible for regulating essential processes in the cells. These free radicals are produced in a variety of metabolic pathways, such as fatty acid oxidation, nucleic acid, and polyamine catabolism. Of note, the discovery of ROS-metabolizing enzymes in peroxisomes has been reported [110], highlighting the importance of peroxisomes in scavenging ROS in cells. 

As mentioned earlier, diabetes causes lipid accumulation. An array of isoforms of PPAR-α, PPAR-γ, and PPAR-σ play an essential role in the regulation of lipid and glucose metabolism. The PPAR-γ isoform is expressed ubiquitously in all tissues [111]. This isoform is the molecular target of a class of insulin-sensitizing drugs known as thiazolidinediones [112]. The PPAR-γ isotypes are capable of reducing inflammatory responses, which are intimately connected to ROS generation in addition to insulin resistance and atherosclerosis. Like mitochondria, peroxisomal ROS/RNS is not considered a hallmark of T2DM. However, it appears that there is a positive correlation between dysfunctional peroxisomes and the elevation of cellular ROS levels [113] and dysregulated lipid metabolism [114]. Since the removal of damaged peroxisomes is not currently possible, antioxidants have been used to maintain a correct redox balance in the cell. The structure and function of peroxisomes and peroxisomal enzymes involved in the production of different ROS are reviewed elsewhere [110]. 

#### 3.1.3. Endoplasmic Reticulum

Hyperglycemia causes endoplasmic reticulum (ER) stress, which has a key role in a number of pathophysiological processes, including endothelial dysfunction, apoptosis in renal cells [115], and pancreatic β-cell failure [116]. The communication networks between ER stress and oxidative stress are by no means unidirectional, as previously described [117].

ER not only regulates the folding and post-translational maturation of a third of all proteins [118] and most secreted proteins, but it also plays a critical role in lipid biosynthesis, detoxification, and reduction-oxidation balance. Numerous endoplasmic reticulum-localized enzymes, including cytochrome p-450 and b5 enzymes and diamine oxidase, contribute to the formation of ROS [119], suggesting the ER has a major influence on maintaining cellular integrity and viability. Another important thiol oxidase enzyme of the oxidative protein-folding cycle, endoplasmic reticulum oxidoreductin 1 (ERO1), catalyzes the transfer of electrons from dithiols to molecular oxygen, resulting in the formation of H_2_O_2_ [120]. During the protein folding process, activation of NOX 4, NADPH-P450 reductase, and glutathione appears to have a causal role in the formation of ROS [121]. The enzymes of ER are responsible for improving protein folding and coping with ongoing oxidative stress. However, the accumulation of unfolded or misfolded proteins within the lumen of the ER causes the induction of ER stress, which impairs normal cellular function via excessive buildup of oxidant H_2_O_2_. Antidiabetic drugs are used as the first line of defense to improve glucose metabolism. Antioxidants, as the second line of defense, that scavenge the active radicals have been considered as an additional molecular mechanism for clinical T2DM treatment.

## 4. Visiting the Most Recent Antioxidants of Medicinal Plants and Their Therapeutic Potential and Mechanisms in Diabetes

**Figure 4:**
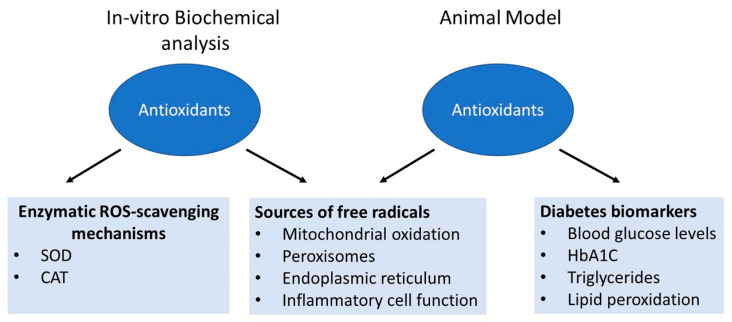
An illustration depicting how the antioxidant properties of polyphenols were measured.

Many plants that possess phenolic compounds are found to have an important protective role in scavenging free radicals during oxidative stress [122]. These studies assess the antioxidant properties of polyphenolic compounds in both in vitro and in vivo models for studying diabetes, as summarized in Figure 4. Here, we provide a brief review of the most frequently used antioxidants to assess their beneficial pharmacological effects on the animal body and humans. 

### 4.1. Flavonoids

Polyphenols are a diverse group of natural compounds that fall into four distinct classes: flavonoids, phenolic acids, stilbenes, and lignans. The effects of the most common polyphenols on the biomarkers of oxidative stress are summarized in Table 1. Flavonoids are the most abundant polyphenols found in an optimal human diet. This class of compounds is further grouped into flavones, flavonols, flavan-3ols, isoflavones, anthocyanidins, and flavanones [123]. Phenolic acids are divided into two sub-groups called hydroxyl benzoic and hydroxyl cinnamic acids. The anti-inflammatory [124] and antioxidant properties of flavonoids [125] have been reviewed (Table 1 and Table 2). Though the mechanisms underlying the benefits of flavonoids are complex and remain incompletely understood, flavonoids have been shown to reduce glycemia and related complications [126,127]. In contrast, others think the bioavailability and effectiveness of flavonoids are relatively low [123]. So, it was suggested that nanoparticle systems should be used to prolong circulation and flavonoid efficacy and potentially reduce their non-specific bindings. 

Seaweed and tropical papaya have been utilized as traditional remedies and passed down through generations. Based on animal research, both papaya leaves and seaweed exhibit beneficial effects in diabetes treatment. They protect against diabetes-induced β cell damage, reduce fasting plasma glucose levels, decrease A1C, increase the expression of antioxidative enzymes, and reduce ROS production [128]. Both seaweed and papaya also contain a package of antioxidants (vitamins A, C, and E complexes) and other substances such as polysaccharides, phenolic compounds, crucial fatty acids, saponins, fucoidans, and phlorotannins found naturally in other fruits and vegetables. It is unlikely that flavonoids alone can accomplish all diabetes outcomes. 

Linarin, a natural flavonoid compound, is capable of counteracting oxidative stress and exerting an anti-inflammatory effect in diabetic mice [129]. Inhibition of aldo-keto reductase (AKR)1B, an NADP(H)-oxidoreductase, by linarin appears to serve as a mechanism for reducing oxidative stress and inflammation in a high-glucose and high-palmitic acid-induced hepatocyte injury model and a T2DM rat model [130].

Isorhamnetin, a methylated derivative of quercetin, belongs to the flavonoid group of phenolic compounds [131]. Isorhamnetin appears to hold great promise against diabetes via aldose reductase inhibition [132]. The important role that isorhamnetin plays in lowering glucose concentrations, improving oxidative status, reducing inflammation, and adjusting lipid metabolism in both in vitro and in vivo models is reviewed [133], suggesting that isorhamnetin may be a useful compound for the treatment of diabetes.

Amomum tsao-ko Crevost et Lemarié (A. tsao-ko), a very common dietary spice, is rich in flavonoids [134]. A. tsao-ko methanol extracts appear to exert remarkable antioxidant and antidiabetic effects in both in vitro and in vivo studies. Nevertheless, further investigations are needed to confirm the detailed mechanisms underlying A. tsao-ko-mediated control of diabetes and oxidative stress. 

With the importance of apigenin in numerous physiological functions, there has been great interest in its strong antioxidant and anti-inflammatory actions [135]. Its action is mediated through neutralizing superoxide, singlet oxygen, and hydroxyl radicals, enhancing the function of the PPARγ signaling, and suppressing CD38 [136,137,138]. 

Licochalcone A, a flavonoid derived from licorice, has been shown to have potential preventative and therapeutic effects on diabetic nephropathy in both in vitro and in vivo studies [139,140]. The antidiabetic effects of the most recent common polyphenols on diabetes are summarized in Table 3.

Myricitrin, a flavone isolated from the bark of *Myrica esculenta*, has demonstrated a substantial reduction in blood glucose levels in type 2 diabetic mouse and rat models [141]. It facilitates glucose absorption by skeletal muscles through the activation of IRS-1/PI3K/Akt/GLUT4 signaling, as evidenced by both in vitro and in vivo studies. Additionally, myricitrin is shown to mitigate oxidative stress by scavenging and neutralizing oxidative radicals and enhancing the body’s natural oxidative defense through nuclear factor erythroid 2-related factor 2 (Nrf-2) activation in both laboratory and live organism studies [142,143].

Biochanin A is a well-known isoflavone for its anti-inflammatory, antihyperlipidemic [144], antioxidant [145], and anti-cancer health benefits [146]. Biochanin A appears to have an anti-diabetic effect beyond its function as an antioxidant. In animal models of T2DM, it causes increased insulin sensitivity [146], reduced glucose tolerance [146], and a reduction in glycohemoglobin A1C formation. Biochanin A maintains steady blood glucose concentrations within a normal range in a T2DM rat model [147]. A meta-analysis shows that increased transforming growth factor-β (TGF-β) levels were associated with a high risk of nephropathy [148]. All 4 subtypes of protease-activated receptors (PAR1-4) are mainly expressed in renal epithelial, endothelial, and podocyte cells [149]. PAR-2 inhibition improves autophagy and prevents fibrosis and inflammation [149]. Surprisingly, biochanin A reduces diabetic nephropathy via suppression of TGF-β1 and PAR-2 gene expression [144,145]. 

Formononetin is an isoflavone from the group of phytoestrogens that induces cell apoptosis via the intrinsic apoptosis pathway, which causes the permeabilization of the mitochondrial outer membrane [150,151]. Formononetin has a wide range of biological activities, including eliciting antioxidant [152] (Table 1) and antidiabetic properties in vitro and in vivo (Table 3). Sirtuin 1 (SIRT1), a nicotinamide adenine dinucleotide (NAD)-dependent histone deacetylase, protects cells from ROS. In addition, SIRT1 regulates hepatic lipid metabolism by increasing AMP-activated protein kinase, leading to the inhibition of hepatic lipogenic pathways in favor of fatty acid oxidation [153]. Formononetin reduces hyperglycemia by increasing SIRT1 expression in pancreatic cells [154]. Sirtuin-induced fatty acid oxidation is important because it lowers cytoplasmic lipid accumulation. However, elevated fatty acid oxidation could interfere with glucose metabolism in the muscle [155]. In addition, formononetin is considered a strong apoptotic inducer [151]. Several other mechanisms of action for formononetin have been described [152,156,157]. It is suggested to be used as adjunct therapy for diabetic neuropathy and nephropathy. Clearly, further research and investigation are needed in order to gain a better understanding of how precisely formononetin improves multiple aspects of metabolic syndrome, including diabetes.

Hesperetin, a derivative of hesperidin, is a bioflavonoid compound found in citrus fruits [158]. Like formononetin, hesperetin has been shown to improve diabetes (Table 3) by regulating SIRT1, alleviating inflammation [158], combating oxidative stress [159], and reducing insulin resistance [159].

Naringenin, a flavonoid compound found in propolis, exhibits potent anti-hyperglycemic and anti-hyperlipidemic properties in diabetic rat models [160]. The actions of naringenin include improving hyperglycemia, insulinemia, insulin sensitivity, pancreatic cell performance, and lipid profile [137,160].

**Table 1:** A summary of the effects of polyphenols in oxidative stress [152,161,162,163,164,165,166,167,168,169,170,171,172,173,174,175,176,177,178,179,180,181,182,183,184,185,186,187,188].

Polyphenols	ROS	SOD	Catalase	NO	Lipid Peroxidase	GSH	RNS	DPPH	Ref.
**Phenolic acids**									
Hexane extract of Eryngium carlinae		NE		↑		NE			[162]
Cinnamic acid	↓	↑				↑		↓	[163,164,165,166]
p-coumaric acid	↓				↓			↓	[167]
Caffeic acid	↓				↓			↓	[168]
Ferulic acid		↑	↑						[169]
Sinapic acid								↓	[170]
Gentisic acid		↓							[171]
Vanillic acid		NE							[171]
Gallic acid	↓				↓			↓	[172]
Syringic acid	↓	↑	↑	↑		↑			[173]
Protocatechuic acid		NE							[171]
**Flavonoids**									
**1.** **van-3-ols**									
Catechin (pumpkin pulp extract)	↓	↑	↑	↑				↓	[174]
Epicatechin									
Epigallocatechin	↓	↑		↑	↓		↓	↓	[175]
**2.** **Isoflavones**	↓	↑				↑			[176]
Biochanin A								↓	[177]
Formononetin		↑	↑			↓			[153]
**3.** **Flavonones**		↑			↓	↓			[153]
Hesperetin									
Naringenin	↓	↑	↑			↑			[179]
**4.** **Flavonols**	↓						↓	WE,↓	[180,181]
Quercetin									
Quercetin + Naringenin								WE	[180]
Kaempferol								↓	[180]
Galangin				↓					[182]
Fisetin	↓	↑	↑						[183]
Myricetin	↓			↓					[184]
**5.** **Flavononol**	↓							↓	[185,186]
Taxifolin									
**6.** **Flavanones**					↓			↓	[187]
Eriodictyol	↓	↑	↑						[188]
**Stilbenes** Resveratrol		↑	↑			↑			[187]
**Phytoestrogen** Lignans								↓	[188]

↑—represents a significantly elevated level; ↓—refers to a significantly reduced level; NE—no effect; WE—denotes weak effect.

Like other flavonoids, kaempferol exerts both antioxidant (Table 1) and anti-inflammatory effects (Table 2). Kaempferol produces anticancer effects through inhibition of epidermal growth factor receptor (EGFR)-dependent Src proto-oncogene, nonreceptor trosin kinase (SRC), ERK1/2, and AKT serine/threonine kinase (AKT) pathways [189], and cleavage of poly(ADP-ribose) polymerase (PARP) [190], an endogenous substrate of caspase [191]. Thus, kaempferol is an antiproliferative, anti-metastatic, and apoptotic drug. Kaempferol exerts an antidiabetic effect (Table 3) through targeting multiple pathways, including improving glycolysis, glucose uptake, glycogen synthesis, AMPK activity, and GLUT4 expression [192]. Additionally, several in vivo studies reported the antidiabetic effect of kaempferol through decreasing plasma glucose level [193], increasing plasma insulin level [193], decreasing glucose synthesis [194], and increasing glucagon-like peptide 1 (GLP-1) and insulin release [195]. Readers are invited to a recent review on all the pharmacological mechanisms of kaempferol in diabetes [196].

While galangin, a flavonoid, exerts antioxidant (Table 1) and anti-inflammatory activity [197,198], it elicits potent antitumor activity in diverse cancers [199]. It is a potent inhibitor of dipeptidyl peptidase-4 (DPP-4) [200], an integral membrane protein expressed in cells. DPP-4 cleaves a large number of bioactive molecules. However, its major physiological substrate is the incretin hormone GLP-1, which is responsible for the maintenance of normal glucose homeostasis [201]. Thus, galangin is an anti-hyperglycemic agent. Although the mechanism of action is not yet completely understood, recent evidence suggests that galangin improves the lipid profile and plasma insulin level [202]. 

Fisetin, a bioactive flavonol molecule, has antiproliferative [203], apoptotic [204], and antioxidant [205] activities (Table 1). Fisetin shows an antidiabetic effect, which is mediated by two different mechanisms [206]. Firstly, fisetin inhibits gluconeogenesis by inhibiting the transport of pyruvate into the mitochondria and reducing the cytosolic NADH/NAD(+) potential redox [206]. Secondly, fisetin inhibits glycogen breakdown, leading to a reduction in blood glucose levels [207,208]. Fisetin represents a promising therapeutic strategy that may synergize with other antidiabetic therapies. 

Myricetin, a flavonoid, is widely distributed in different types of fruits, herbs, and tea [209]. Recent studies have reported myricetin’s mechanisms of action in diabetes, such as inhibiting DPP4 [210], being an inactivator of GLP-1, or serving as a GLP-1 receptor agonist [211]. Surprisingly, myricetin appears to normalize the intestinal flora of type 2 diabetic mice [212].

Anthocyanins, polyphenolic compounds of the flavonoid group, regulate digestive enzymes (α-amylase and α-glucosidase), GLUT-4, GLP-1, glucose-6-phsphatase (G6Pase), phosphoenolpyruvate carboxykinase (PEPCK), or PPARγ. These compounds also manage blood glucose levels by normalizing insulin secretion and insulin resistance [213]. Additional significant mechanisms involve the protection of pancreatic β cells through their anti-inflammatory and antioxidant properties [214]. It is unlikely that anthocyanins alone can regulate various classes of enzymes. Further investigations are required to confirm the clinical utility of anthocyanins for the treatment of diabetes, and studies should be standardized and quantified to draw universal conclusions regarding their true use, as suggested by the authors [213].

The extract of *Delonix regia* shows hypoglycemic, antioxidant, and hypolipidemic properties [215]. Of note, the antidiabetic effect of this extract was found to be similar to that of glibenclamide, a well-known antidiabetic agent, which lowers blood glucose levels by stimulating the amount of insulin produced by the pancreas. However, the constituents in Delonix regia extracts that exert antioxidant, hypoglycemic, and adverse effects remain uncharacterized. 

Mulberry (*Morus alba* L.) leaves, used extensively as an effective traditional Chinese medicine for blood glucose management, have been reported to have beneficial effects on skeletal muscle function [216]. It appears that the flavonoids present in mulberry leaves markedly ameliorate skeletal muscle insulin resistance and enhance mitochondrial function in diabetic mice through the AMPK-PGC-1α signaling pathway [216,217,218]. Flavonoids in mulberry leaves show hypoglycemic effects via inhibiting the TGF-β1 pathway [219] and increasing antioxidase activity. Although very interesting, more randomized controlled trials should be required to warrant the findings of this study.

Vaccarin (VAC), a potent flavonoid glycoside extracted from Vaccariae Semen, has been discovered to improve blood glucose levels and insulin resistance, reduce oxidative stress, and enhance endothelium-dependent vasodilation in T2DM mouse models [220,221] (Table 1). Thus, *Vaccariae Semen* may serve as an inhibitor of ROS and RNS, which are instigators of several illnesses, as mentioned earlier (Table 2). It was further shown that vaccarin improves glucose metabolism and vascular endothelial function through the inhibition of the ROS/AMPK/miRNA-34a/eNOS signaling cascade [220]. The protective effects of vaccarin against ROS and RNS as a dietary supplement could be used as adjuvant therapy for T2DM. 

Recent research has shown that the flavonoid extract of fenugreek restores the antioxidant enzyme activities of both SOD and catalase and causes a decrease in malondialdehyde content [222]. This extract also alleviates hyperglycemia in a streptozotocin (STZ)-induced T2DM mouse model. 

Taxifolin, a recognized flavonoid, is found to be most active towards in inhibiting α-amylase. Thus, taxifolin has the ability to manage post-meal hyperglycemia [223]. It also prevents diabetic cardiomyopathy via inhibition of oxidative stress [224]. This function, in conjunction with its anti-inflammatory and antioxidant properties, can contribute to the treatment of diabetes mellitus.

A flavonoid-rich fraction derived from *Trichilia emetica* exhibits prominent radical scavenging and antidiabetic activities [225]. The binding of free ferrous to oxygen yields ferric iron and superoxide, thus generating hydrogen peroxide. Formed hydrogen peroxide reacts with ferrous iron and gives rise to hydroxyl radical formation. *Trichilia emetica* flavonoid-rich fractions are found to be capable of chelating ferrous ions. Although this plant could potentially be a valuable agent exhibiting strong antioxidant activity for controlling high blood sugar levels, the constituents in *Trichilia emetica* extracts remain uncharacterized.

2,3-dihydroxybenzoic acid (DHBA), a phenol, is a weak iron chelator and radical scavenger [226] (Table 2). This colonic-derived flavonoid metabolite is found to regulate glucose uptake and production in renal tubular NRK-52E cells [227]. Additional studies are needed to confirm whether or not this phenol offers a treatment benefit in a reliable large animal diabetes model.

**Table 2:** A summary of polyphenol-mediated protection against inflammation and associated oxidative stress [152,228,229,230,231,232,233,234].

Polyphenols	ROS	NOX-4	ICAM-1	p-ERK	VCAM	TNF-α	IL-6	MCP-1	MAPKs	Metal ion Chelator	Ref.
**Phenolic acids** Epicatechin (EC) 2,3-dihydroxybenzoic acid (DHBA)	↓ ↓	↓ ↓	↓ ↓	↓ ↓	↓ ↓	↓ ↓	↓ ↓	↓ ↓	↓ ↓		[228] [228]
**Flavonoids** Catechins Formononetin	↓	↓				↓ ↓	↓			↓	[229] [152]
**Stilbenes** Resveratrol	↓		↓		↓	↓	↓		↑		[230,231,232]
**Phytoestrogens** Lignans Isoflavone				↓ ↓							[233] [234]

↑ represents a significantly elevated level; ↓ refers to a significantly reduced level.

**Table 3:** A summary of polyphenols as biomarkers of oxidative stress in diabetes [152,161,235,236,237,238,239,240].

Polyphenols	Glucose	A1C	Water Intake	Volume of Urine	NO_2_^−^/NO_3_^−^	Heme Oxygenase-1 (HO-1)	Nrf2	Insulin Resistance	Lipoperoxidation	Ref.
**Phenolic acids** Phenethyl ester of caffeic acid Ferulic acid (FA) Hexane extract of *Eryngium carlinae*	↓ ↓		↓	↓	↓	↓	↑		↓	[235] [236] [161]
**Flavonoids** Catechins Formononetin	↓ ↓		↓	↓				↓		[237] [152]
**Stilbenes** Resveratrol						↑	↑			[238]
**Phytoestrogen** Lignans	↓	↓	↓						↓	[239,240]

↑ represents a significantly elevated level; ↓ refers to a significantly reduced level.

### 4.2. Catechins

Catechin, a flavonoid, is found in a variety of foods and herbs, including tea. As direct antioxidants, catechins are ROS scavengers and metal ion chelators (Table 1) [241]. As indirect antioxidants, they induce the activation of antioxidant enzymes [229], although catechins become pro-oxidants at micromolar levels in cells supplemented with iron and linoleic acid [241]. The antioxidant enzyme pathway is a key player in maintaining the delicate balance between ROS and RNS generation and antioxidants in both animal cells and plant cells. Antioxidant enzyme levels do not appear to be significantly changed in various tissues of aging animals [242,243,244]. However, stimulating this pathway to activate the expression [245] and the delivery of catalytic mimetic of the antioxidant enzyme pathway [246] has been considered important for preventing diseases caused by oxidative stress and various exogenous substances [247,248,249]. Various pumpkin species contain large amounts of catechin and kaempferol [250]. The pumpkin pulp shows antioxidant effects based on DPPH radicals and ferric-reducing antioxidant power (FRAP) radicals. However, the antioxidant potential of these polyphenols cannot be estimated based on the content of individual bioactive compounds, as described in detail in the paper. 

Epigallocatechin Gallate (EGCG), a sub-class of flavonoids, is an antioxidant (Table 1). EGCG is identified not only as a potential autophagy regulator, but it is also capable of diminishing the expressions of inflammation-associated genes in peripheral leukocytes and adipose tissue of non-obese type 2 diabetic Goto-Kakizaki rats [225,251,252]. Evidence indicates that EGCG has pro-oxidant activity due to its instability and autoxidation [253].

Procyanidins are oligomeric compounds composed of catechin and epicatechin molecules. Research suggests that plants rich in procyanidins could have preventive effects on hyperglycemia and T2DM [254]. The readers can find additional information, including the structure, classification, and underlying mechanisms of catechins in regulating diabetes, by Wen et al., 2022 [237]. 

Although catechins have been considered beneficial in protecting against various diseases caused by oxidative stress and ROS, such as CVD and diabetes [237], and in hematology [173], their effects on CVD biomarkers such as the levels of C-reactive protein, B-type natriuretic peptide, N-terminal pro-atrial natriuretic peptide, aldosterone, renin, fibrinogen, D-dimer, plasminogen-activator inhibitor type 1, homocysteine, and the urinary albumin-to-creatinine ratio are not fully assessed.

### 4.3. Phytoestrogens

Lignan, a phytoestrogen, and polyphenols isolated from Linum usitatissimum show antidiabetic properties in STZ-induced diabetic rats [239]. Daily treatment with flaxseed extract improves HbA1c levels and blood glucose levels. It also reduces the total cholesterol, high-density lipoprotein (HDL), LDL, and triglyceride levels significantly in diabetic rats.

## 5. Clinical Trial of Antioxidant Therapy in Patients with Diabetes 

Inflammation is intimately tied to higher glycated hemoglobin (HbA1c) levels, a good indicator of chronic hyperglycemia. Setting the treatment target for HbA1c below 6% in high-risk diabetic patients leads to reduced 5-year nonfatal myocardial infarctions in the Diabetes (ACCORD) Study Group. A new model of treating patients with antioxidants with inflammatory actions is emerging as a promising therapeutic option for patients with T2DM. The evidence provided by these various studies supports that ginger, resveratrol, and rutin flavonoid could reduce the HbA1c level, blood glucose level, and homeostatic model evaluation of insulin resistance (HOMA)-IR, an indicator of long-term glycemic control, while allium sativum, olea europaea oil, and astaxanthin cause a decrease in LDL and serum total cholesterol (Table 4). 

Mudan granules possess neuroprotective effects in diabetic patients. Apparently, there is a new opportunity for these antioxidants and anti-inflammatory agents like resveratrol and rutin flavonoid to demonstrate their activity in combination with mudan granules. Notably, while ginger causes an increase in NO production, resveratrol and rutin flavonoid reduce CRP and IL-6, respectively. Thus, antioxidants may be effective for the treatment of cytokine storms in diabetes. The fact that these compounds were able to pass preclinical research and enter human studies indicates their potential effectiveness. The above data show the clinical utility of antioxidants with anti-inflammatory effects in T2DM. However, the application of these antioxidants requires further research investigations to evaluate their efficacy against T2DM-related complications, including hyperlipidemia, pro-inflammatory mediators, and insulin insensitivity. There is also a need to select diabetic patients at various stages of the disease to guarantee that the anti-inflammatory and antidiabetic effects of the compound are evident.

## 6. Conclusions and Future Implications

ROS mediates many important signaling functions in different cellular and developmental processes in human cells. The level and sources of intracellular ROS are critically important for their actions in influencing different complex signaling and biochemical pathways under both physiological and pathophysiological conditions. 

Diabetes, a metabolic disorder, is associated with reduced levels of antioxidants, which can reduce the susceptibility of pancreatic islets to oxidative stress. Oxidative stress impairs insulin-mediated intracellular signaling pathways. Moreover, while insulin resistance is a complex metabolic disorder, oxidative stress can induce insulin resistance. 

Diabetes triggers changes in endothelial function, which may lead to increased oxidative stress mediators and a reduction in antioxidants. Oxidative stress can be the result of mitochondrial dysfunction or inflammation, not the cause. Evidence indicates that oxidative stress accelerates the development of complications in diabetes. The diseases that accompany T2DM include myocardial infarction, stroke, peripheral artery disease, angina pectoris, and periodontal disease, a widespread condition that affects a large percentage of the population. These pathologies seem to arise from the same risk factors and display a similar inflammation profile. Identifying novel biomarkers and therapeutic strategies to prevent diabetes-induced endothelial dysfunction is of great interest. 

Chronic production of both proinflammatory and proatherogenic mediators leads to systemic inflammatory diathesis, causing a decrease in beta cell insulin secretion and an increase in insulin resistance. The resulting chronic hyperglycemia and hyperlipidemia promote the inflammatory process, leading to a vicious cycle. Antidiabetic drugs such as metformin, pioglitazone, insulin, glucagon-like peptide-1 agonists, and dipeptidyl peptidase-4 inhibitors are found to reduce inflammation. Statin is capable of ameliorating inflammation to some extent. While anti-inflammatory drugs such as IL-1 and TNF-α antagonists are in trials, salsalate, an NSAID, appears to improve insulin sensitivity. Anti-inflammatory drugs are associated with a broad range of side effects, such as cardiovascular events and the worsening of congestive heart failure. Sirtuin and 12-lipo-oxygenase are considered new therapeutic targets for T2DM and inflammatory diseases. Unfortunately, some of the antidiabetic and anti-inflammatory drugs are linked to renal toxicity. The prevalence of total diabetes is expected to increase over the next ten years. More than ever before, it is imperative that research focus on developing new anti-inflammatory drugs with improved efficacy and safety as well as antioxidants, which serve as “safety switches” to prevent unwanted inflammatory responses via neutralizing free radicals. In this review, we highlighted that a number of therapeutic medicines derived from plants are promising sources of natural antioxidants and exhibit anti-inflammatory activities. 

The United Kingdom Prospective Diabetes Study clearly verifies the importance of glycemic control in reducing the risk of microvascular complications. While the efficacy of antioxidative treatments remains elusive, recent clinical reports indicate that antioxidants have the ability to counteract inflammation and improve hyperglycemia, as well as beneficial effects on hepatocyte lipid metabolism through different mechanisms in diabetes. The mechanisms by which the antioxidants reduce hyperglycemia are only partly known. Since diabetes is a progressive disease, T2DM patients’ need for different treatments changes as well. It remains to be shown whether antioxidant treatments administered together with current antidiabetic or anti-inflammatory drugs can prevent the vascular complications of diabetes. Further studies are required to clarify the role of antioxidant therapy in the management of type 2 diabetes.

## Figures and Tables

**Table 4:** Summary of the acute effects of supplemental dietary antioxidant therapy for the prevention and treatment of diabetes in a double-blind placebo-controlled study [255,256,257,258,259,260,261,262].

Dietary Source	Dose (Daily)	T2DM Patients	Inflammatory and Oxidative Stress Markers	Diabetes Markers	Cardiovascular Risk Markers	Renal Markers	Ref.
Allium sativum and Olea europaea oil		160 patients		Decreased serum cholesterol, triglycerides (TGs), and low-density lipoprotein (LDL) Increased High-density lipoprotein (HDL) levels			[255]
Astaxanthin	12 mg	24 patients		Not a significant change in glucose and insulin levels Decreased low-density lipoprotein and total cholesterol	Reduced fibrinogen, L-selectin, and fetuin-A		[256]
Beetroot juice (concentrated)	24 ml	46 patients		No effect			[257]
Ginger	2000 mg	44 hemodialysis patients with end-stage renal disease	A higher nonsignificant increase nitric oxide	Reduced blood glucose levels, decreased homeostatic model evaluation of insulin resistance (HOMA)-IR		Decreased creatinine	[258]
Mudan granules	3 times	93 patients with painful diabetic neuropathy	Reduced pain and numbness in the extremities Increased nerve conduction velocity				[259]
Resveratrol	1000 mg	97 older adults with T2D	Reduced lipoperoxides, isoprostanes and C-reactive protein levels	HbA1c level Hypolipemic effect			[260]
Resveratrol plus delta-tocotrienol	400 mg capsule (delta-tocotrienol 250 mg; resveratrol 150 mg)	82 patients with metabolic syndrome	Reduced C-reactive protein, interleukin-6, tumor necrosis factor-alpha, malondialdehyde	Reduced fasting plasma glucose, serum triglyceride			[261]
Rutin flavonoid		50 patients	Decreased interleukin 6, total antioxidant capacity, and malondialdehyde	Decreased FBG, insulin, HbA1c, homeostasis model assessment of insulin resistance (HOMA)-IR; Decreased LDL-c, triglyceride (TG)			[262]

## Data Availability

The original contributions in the study are included in the article, further inquiries can be directed to the corresponding author.

## Abbreviations

A. tsao-ko	Amomum tsao-ko Crevost et Lemarié
AGE	Advanced glycation end
AKR	Aaldo-keto reductase
AKT	Protein kinase
AMPK	AMP-activated protein kinase
ATP	Adenosine triphosphate
CAT	Catalase
CDC	Centers for Disease Control and Prevention
CRP	C-reactive protein
CVD	Cardiovascular diseases
DHBA	2,3-dihydroxybenzoic acid
DPP-4	Dipeptidyl peptidase-4
EGCG	Epigallocatechin Gallate
EGFR	Epidermal Growth Factor Receptor
ER	Endoplasmic reticulum
ERK1/2	Extracellular Signal-Regulated Kinase 1/2
ERO1	Endoplasmic reticulum oxireductin 1
FRAP	Ferric reducing antioxidant power
G6Pase	Glucose-6-phosphatase
GLP-1	Glucagon-like peptide 1
GLUT4	Glucose transporter 4
GPx	Glutathione peroxidase
H_2_O_2_	Hydrogen Peroxide
HbA1c	Hemoglobin A1c
HDL	High-density lipoprotein
HOMA-IR	Homeostatic model evaluation of insulin resistance
IL-1	Interleukin-1
IL-1β	Interleukin-1 beta
IL-6	Interleukin-6
IRS-1	Insulin Receptor Substrate 1
LDL	Low-density lipoprotein
MAPK	Mitogen-activated protein kinase
mDNA	Mitochondrial DNA
NAD	Nicotinamide Adenine Dinucleotide
NADH	Nicotinamide Adenine Dinucleotide
NADPH	Nicotinamide adenine dinucleotide phosphate
NF-kB	Nuclear factor kappa B
NHANES	National Health and Nutritional Examination Survey
NO	Nitric Oxide
NOXs	Nicotinamide adenine dinucleotide phosphate oxidase
PAR	Protease-activated receptor
PARP	Poly(ADP-Ribose) Polymerase
PEPCK	Phosphoenolpyruvate carboxykinase
PGC-1α	Peroxisome Proliferator-Activated Receptor Gamma Coactivator 1 alpha
PI3K	Phosphoinositide 3-Kinase
PKC	Protein kinase C
PPAR α	Peroxisome proliferator-activated receptor alpha
PPAR γ	Peroxisome proliferator-activated receptor gamma
RAGE	Receptor for advanced glycation end products
RNS	Reactive nitogen species
ROS	Reactive oxygen species
SDG	Secoisolariciresinol diglucoside
SIRT1	Sirtuin1
SOD	Superoxide dismutase
STZ	Streptozotocin
T2DM	Type 2 diabetes mellitus
TGF-β	Transforming growth factor-beta
VAC	Vaccarin
